# Use of a Single Hybrid Imaging Agent for Integration of Target Validation with *In Vivo* and *Ex Vivo* Imaging of Mouse Tumor Lesions Resembling Human DCIS

**DOI:** 10.1371/journal.pone.0048324

**Published:** 2013-01-11

**Authors:** Tessa Buckle, Joeri Kuil, Nynke S. van den Berg, Anton Bunschoten, Hildo J. Lamb, Hushan Yuan, Lee Josephson, Jos Jonkers, Alexander D. Borowsky, Fijs W. B. van Leeuwen

**Affiliations:** 1 Department of Radiology, Interventional Molecular Imaging Laboratory, Leiden University Medical Center, Leiden, The Netherlands; 2 Departments of Radiology and Nuclear Medicine, Netherlands Cancer Institute- Antoni van Leeuwenhoekhuis, Amsterdam, The Netherlands; 3 Department of Radiology, Leiden University Medical Center, Leiden, The Netherlands; 4 Center for Molecular Imaging Research, Massachusetts General Hospital and Harvard Medical School, Charlestown, Massachusetts, United States; 5 Division of Cell Biology, Netherlands Cancer Institute–Antoni van Leeuwenhoek Hospital, Amsterdam, The Netherlands; 6 Department of Pathology and Laboratory Medicine, Center for Comparative Medicine, School of Medicine, University of California at Davis, Sacramento, California, United States of America; National Institute of Health, United States of America

## Abstract

Screening of biomarker expression levels in tumor biopsy samples not only provides an assessment of prognostic and predictive factors, but may also be used for selection of biomarker-specific imaging strategies. To assess the feasibility of using a biopsy specimen for a personalized selection of an imaging agent, the chemokine receptor 4 (CXCR4) was used as a reference biomarker.

**Methods:**

A hybrid CXCR4 targeting peptide (MSAP-Ac-TZ14011) containing a fluorescent dye and a chelate for radioactive labeling was used to directly compare initial flow cytometry–based target validation in fresh tumor tissue to *in vivo* single photon emission computed tomography (SPECT) imaging and *in vivo* and *ex vivo* fluorescence imaging.

**Results:**

Flow cytometric analysis of mouse tumor derived cell suspensions enabled discrimination between 4T1 control tumor lesions (with low levels of CXCR4 expression) and CXCR4 positive early, intermediate and late stage MIN-O lesions based on their CXCR4 expression levels; CXCR4^basal^, CXCR4^+^ and CXCR4^++^ cell populations could be accurately discriminated. Mean fluorescent intensity ratios between expression in MIN-O and 4T1 tissue found with flow cytometry were comparable to ratios obtained with *in vivo* SPECT/CT and fluorescence imaging, *ex vivo* fluorescence evaluation and standard immunohistochemistry.

**Conclusion:**

The hybrid nature of a targeting imaging agent like MSAP-Ac-TZ14011 enables integration of target selection, *in vivo* imaging and *ex vivo* validation using a single agent. The use of biopsy tissue for biomarker screening can readily be expanded to other targeting hybrid imaging agents and can possibly help increase the clinical applicability of tumor-specific imaging approaches.

## Introduction

Screening of biomarker expression levels in breast cancer biopsy samples using immunohistochemistry (IHC) is a routine procedure that provides an assessment of prognostic and predictive factors such as histological grade, subtype and hormone receptor and human epidermal growth factor receptor 2 (Her2/neu) status [1], [2]. The molecular insights derived from these biopsy samples can be used for decision-making in (personalized) treatment planning. For example, estrogen receptor (ER) and/or the Her2/neu status in biopsy samples can predict the response to trastuzumab when added to standard cytotoxic adjuvant chemotherapy [3]–[5]. Additionally, staining of biopsy tissue for less established biomarkers such as the chemokine receptor 4 (CXCR4) has been shown to correlate with aggressiveness/invasiveness and metastatic potential in breast cancer [6]–[8].

The current standard of care in (preoperative) non-invasive imaging of breast cancer includes implementation of contrast enhanced MRI and ^18^F-FDG PET. Both modalities are widely applied in the detection of cancer and many other diseases. They rely on differences in perfusion/vascular “leakiness” (MRI) and metabolism/glucose uptake (PET) between diseased and normal tissue. For more specific visualization of e.g. tumor tissue, at present, numerous alternative imaging agents are being developed which directly target specific biomarkers expressed on the cell membrane.

Expression patterns of such biomarkers tend to be heterogeneous and vary between patients and tumor subtypes, which could also imply the need for more than one targeting compound for accurate imaging-based assessment of a specific tumor lesion. However, realistically, one cannot perform consecutive biomarker screening studies in a single patient. Similar to their use in treatment selection, individual biomarker expression patterns may also be exploited for specific imaging strategies, as was shown by Dijkers et al. who performed non-invasive positron emission tomography (PET) imaging of Her2/neu positive lesions in patients with metastatic breast cancer [9].

**Figure 1:**
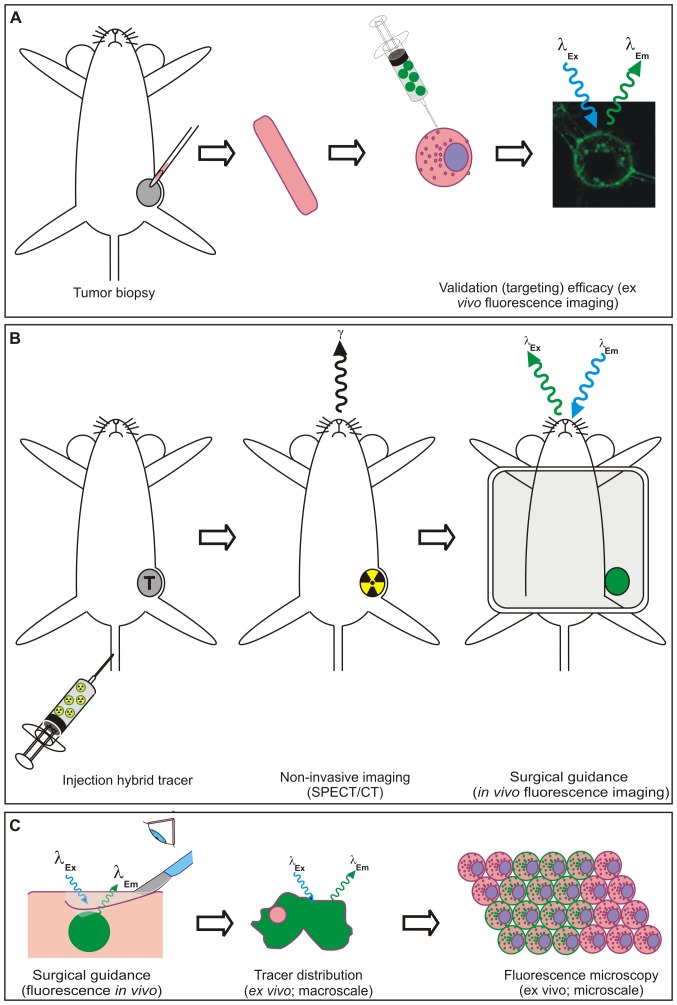
Schematic representation of the integrated logistics made possible by using a targeting hybrid imaging agent. A) Analysis of tumor biopsy samples using the fluorescent beacon of the imaging agent using flow cytometry. B) Non-invasive tumor visualization using SPECT/CT after radiolabeling of the hybrid agent. Fluorescence imaging enables intraoperative surgical guidance. C) *Ex vivo* evaluation of tracer distribution using fluorescence imaging and –microscopy after excision of the tumor.

Identification of a biomarker or rather a diagnostic target during the different logistical steps in clinical management viz. IHC of biopsy tissue, (preoperative) imaging, intraoperative surgical guidance and pathological evaluation of resection margins, are all commonly performed using different methods and different (targeting) compounds. This variation may lead to a discrepancy in findings. In an ideal situation, however, target selection and further follow-up are conducted using one and the same imaging agent. This should yield more interchangeable and complementary results during the whole logistical process of cancer management (Fig. 1). For this reason a “smart” screening method for an imaging approach or a combination thereof is required.

We have recently demonstrated the clinical value of hybrid tracers. The hybrid tracer ICG-^99 m^Tc-nanocolloid, enables both the diagnostic identification of sentinel lymph nodes (radioactive component; ^99 m^Tc) and provides optical guidance during the surgical resection (fluorescent component; ICG) [10]–[15]. This hybrid surgical guidance approach has already been applied in over 300 patients and for a number of different tumor locations. Integration of this concept with a (tumor) targeting moiety will aid in the resection of primary tumors and metastases [16].

For a targeted imaging approach a tailored selection process that identifies the best diagnostic target will be instrumental for the successful application of biomarker specific imaging agents. With this in mind we reasoned that a biopsy specimen can potentially be used for the selection of a specific imaging agent.

To demonstrate the feasibility of integrating biopsy screening in fresh breast tumor tissue with in vivo imaging, the chemokine receptor 4 (CXCR4) was used as a reference biomarker. In a recent critical review, in which we evaluated CXCR4 targeting imaging agents based on their affinity, specificity and biodistribution, the T140 peptide derivative Ac-TZ14011 was shown to be one of the best targeting moieties for evaluation of CXCR4 expression levels using fluorescence imaging [17]. Different imaging labels on the Ac-TZ14011 peptide have been shown to aid the specific visualization of CXCR4 expressing tumor cells with: i) fluorescence IHC (FITC labeled version), ii) flow cytometric analysis (FITC labeled version), iii) SPECT/CT (^111^In-DTPA labeled version) and iv) *in vivo* fluorescence imaging (near-infrared labeled version) [18]–[20]. The synthetic development of a hybrid version of this targeting peptide (MSAP-Ac-TZ14011), which contains both a fluorescent label and a chelate for radioactive labeling, enabled integration of *in vitro* affinity evaluation and *in vivo* imaging methods [21], [22].

In this study the concept of using a biopsy specimen for a personalized selection of the most optimal targeting imaging approach was evaluated using MSAP-Ac-TZ14011. The fluorescent label was used to assess the membranous CXCR4 expression pattern in fresh tumor segments obtained from tumor bearing mice. After radioactive labeling with 111-indium, the same imaging agent was suitable for in vivo SPECT/CT imaging. The initial screening results obtained with flow cytometry (Fig. 1A) were directly correlated to *in vivo* imaging results (SPECT/CT and fluorescence imaging; Fig. 1B), and microscopic *ex vivo* analysis (fluorescence confocal microscopy; Fig. 1C); hereby comprising all steps in clinical cancer management.

## Materials and Methods

### Synthesis and radiolabeling of MSAP-Ac-TZ14011

**Figure 2:**
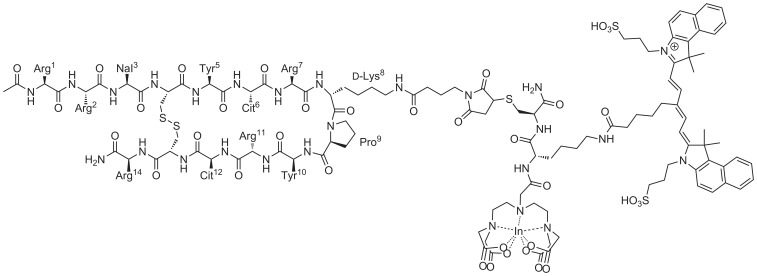
Structure of MSAP-Ac-TZ14011.

MSAP-Ac-TZ14011 (Fig. 2) was synthesized and radiolabeled according to previously described procedures [22].

### *In vivo* model

As a CXCR4 positive tumor model the orthotopic MIN-O transplantation model resembling human ductal carcinoma in situ was used [23], [24]. In this model, preinvasive lesions progress into invasive lesions with increasing membranous CXCR4 expression [19]. Tumor lesions were staged according to previously reported criteria; based on CT-based size measurements and IHC discrimination was made between early stage (<100 mm^3^), intermediate stage (100–400 mm^3^), and late stage (>400 mm^3^) lesions [19].

Cell line based 4T1 tumor lesion served as control. In these control tumors, CXCR4 expression is constantly low during tumor progression [19] and therefore no discrimination between stages was made. 4T1 cells (from American Type Culture Collection, ATCC) were a kind gift of dr. O. van Tellingen, NKI-AvL, Amsterdam, The Netherlands.

All tumor lesions were generated as reported previously [19]. Animal experiments were performed in accordance with Dutch welfare regulations and were approved by the ethics committee of the Netherlands Cancer Institute under references 08021 B19 and 08021 B21. Implantation of tumor tissue or cells and in vivo imaging were performed under hypnorm/dormicum/water (1∶1∶2; 5 µL/g i.p.) anesthesia. All efforts were made to minimize suffering.

### Fresh tumor tissue analysis

From fresh tumor specimens (MIN-O: n = 6 per stage; 4T1: n = 6) single cell suspensions were made by cutting the tumor into small pieces with a scalpel and suspending them using a 18G, 21G and a 25G needle, respectively. The cell suspension was incubated for 5 minutes with an ER-lysis buffer (0.31 M NH_4_Cl, 0.02 M KHCO_3_, 0.5 M EDTA in 2 L H_2_O; pH 7.4) to remove red blood cells. 300,000 cells per measuring condition were washed with 0.1% bovine serum albumin in phosphate buffered saline (0.1% BSA/PBS) and incubated for 1 hour at 4°C under dark conditions with MSAP-Ac-TZ14011 (1∶200 from a stock of 1 mg/mL) or with the monoclonal phycoerythrin (PE) labeled anti-CXCR4 antibody 2B11 (2B11-PE; 1∶100; BD Biosciences). For evaluation of the overlap in staining between MSAP-Ac-TZ14011 and 2B11-PE, cells were co-incubated with MSAP-Ac-TZ14011 and 2B11-PE.

Following incubation cells were washed with 0.1% BSA/PBS and propidium iodide (PI; 1∶10.000; BD Biosciences) was added to allow the selection of viable cells. Antibodies were diluted in 0.1% BSA/PBS in all flow cytometric experiments. Non-peptide/antibody incubated cells served as controls. Cells were analyzed (approximately 20,000 events per sample) using a CyAn ADP flowcytometer (DakoCytomation) equipped with Summit v4.3 sorftware (DakoCytomation). PE fluorescence was detected after excitation at 488 nm. Emission was collected at 575/25 nm. PI was detected after excitation at 488 nm and emission was collected at 613/20 nm. The CyAL-5.5_b_ dye on the MSAP label was exited at 635 nm and emission was collected at 665/20 nm. Cell viability was comparable between samples.

For evaluation of CXCR4 staining, stained populations were divided into CXCR4^−^ (CXCR4 negative cells), CXCR4^basal^ (basal/low expression of CXCR4), CXCR4^+^ and CXCR4^++^, based on the measured cell surface associated fluorescence; populations were discriminated based on differences in the mean fluorescence of that specific population. Mean fluorescence intensity ratios (MFIR) were calculated by dividing the mean fluorescence intensity of all cells stained by MSAP-Ac-TZ14011 by the mean fluorescence intensity of the non-incubated control. The ratio between the MIN-O and 4T1 tumor lesion was determined by dividing the MFIR of the various MIN-O tumor lesions by MFIR of the 4T1 tumor lesion. The ratio between the CXCR4^basal^ and the CXCR4^+^ and CXCR4^++^ populations was determined by calculating the MFIR between the CXCR4^+^ or CXCR4^++^ and the CXCR4^basal^ population. This results in a semi quantitative evaluation of the level of over-expression.

To evaluate the amount of lymphocytes in the CXCR4 positive population in the tumor cell suspension, cells were co-incubated with MSAP-Ac-TZ14011 and the PE-labeled anti-CD45 antibody (CD45-PE; 1∶200; eBioscience). CXCR4 positive lymphocytes were defined as cells that were both CXCR4 positive (CXCR4^basal^, CXCR^+^ and/or CXCR4^++^) and CD45 positive. The total percentage of CXCR4 positive lymphocytes was determined using the following formula: (CD45^+^ population within the CXCR4 positive population / total amount of CXCR4 positive cells)×100%. The different CXCR4 positive populations were selected and the presence of lymphocytes in each population was determined in a similar manner as used for the whole population: (CD45^+^ population within the selected CXCR4 positive population / total amount of cells in the selected population)×100%. Statistics were performed using a standard T-test.

### Confocal imaging of fresh tumor slices

For direct *ex vivo* evaluation of CXCR4 staining, 4T1 (n = 3) and late stage MIN-O (n = 3) tumor bearing mice were sacrificed and the tumor was removed. Next, the tumor was cut into thin tissue slices which were then incubated with MSAP-Ac-TZ14011 (1∶200 in MEM medium) for 1 hour at 4°C under dark conditions.

For comparison, 4T1 (n = 3) and late stage MIN-O (n = 3) tumor bearing mice were intravenously injected with 50 µg MSAP-Ac-TZ14011. Twenty-four hours after injection, mice were sacrificed where after the tumor was removed and cut into thin slices.

Before analysis using the Leica TCS SPII AOBS confocal microscope (Leica Microsystems), slices were incubated with DAPI, washed thoroughly with PBS and placed on 24 mm ø glass coverslips. Non-incubated tumor slices were used as negative control. Images were acquired at 37°C following excitation at 633 nm at 10× and 63× magnification. Emission was collected from 650–725 nm. DAPI was excited at 405 nm and emission was collected from 409–468 nm. Images were analysed using Leica Confocal Software (Leica Microsystems).

### Immunohistochemistry (IHC)

Formalin fixed paraffin embedded MIN-O or 4T1 tumor tissue sections were stained according to the protocol previously reported by van den Berg et al. [18] with a monoclonal anti-CXCR4 antibody (Rat-anti-CXCR4 clone 2B11 1∶100; BD Biosciences). Images were obtained at 40× magnification. Membranous staining was assessed as previously reported [19]. The ratio between the MIN-O and 4T1 tumor lesion was determined by dividing the percentage of membranous staining in the MIN-O tumor lesions by the percentage of membranous staining in the 4T1 tumor lesions. Statistics were performed using a standard T-test.

### *In vivo* imaging

Tumor bearing mice (n = 5 for intermediate stage MIN-O; 100–400 mm^3^) lesions and n = 5 for late stage 4T1 tumor lesion (<400 mm^3^)) were injected intravenously with 50 µg ^111^In-MSAP-Ac-TZ14011 (10 MBq). SPECT/CT scans were conducted on a preclinical SPECT/CT scanner (Nanospect; Bioscan) 24 hours post injection. After acquisition, the CT data was reconstructed using a cone-beam filtered back projection and SPECT data were reconstructed iteratively with HiSPECT software (Scvis GmbH). Signal intensities were analyzed using the InVivoScope post-processing software (Bioscan Inc.). For further details, see van Leeuwen *et al.* [25]. After SPECT/CT imaging, mice were sacrificed. Tumor-to-muscle ratios were determined after measurement of radioactivity as previously reported [19].

*In vivo* fluorescence imaging was conducted on the IVIS 200 camera (Xenogen Corp.) using Living Imaging Acquisition and Analysis software (Xenogen Corp.). Images were acquired with standard Cy5.5 (excitation 615–665 nm and emission 695–770 nm) settings. Fluorescent content was measured in photons/sec/cm^2^.

## Results

To set up an analytical method that can be applied for screening of fresh biopsy specimens, cell suspensions of freshly obtained tumor segments were prepared. After incubation of the tumor derived cell suspensions with MSAP-Ac-TZ14011, flow cytometric analysis revealed differences in fluorescent intensity levels between the samples.

### CXCR4 expression levels in MIN-O and 4T1 tumor lesions

In the MIN-O tumor lesions the mean fluorescence intensity ratio (MFIR) of all stained cells increased from 165.5±13.8 in early stage to 367.9±22.4 in late stage MIN-O lesions (for MFIR values see Table 1). Overall, the MFIR found in the MIN-O lesions is 3-fold (range 1.8–4) higher than in the 4T1 tumor lesions (MFIR 91.5±14.4; p<0.001; Table 1). This result is comparable to the ratios found between MIN-O and 4T1 control tumor lesions after flow cytometric assessment using the anti-CXCR4 antibody 2B11-PE [19]. Comparable ratios were also found when comparing ratios found with MSAP-Ac-TZ14011 based flow cytometry (Table 1) to quantified membranous staining (IHC) in MIN-O and 4T1 tumor lesions.

**Table 1:** Evaluation of CXCR4 expression with flow cytometric analysis and IHC.

	MFIR CXCR4^+^ and CXCR4^++^ population	Ratio MFIR MIN-O/4T1	Ratio membranous staining MIN-O/4T1 (ex vivo)
MIN-O (early stage)	165.5±13.8	1.8	1.1
MIN-O (intermediate stage)	329.6±33.6	3.6	3.5
MIN-O (late stage)	367.9±22.4	4.0	5.5
4T1	91.5±14.4	-	-

For MIN-O tumor tissue, 6 biopsy samples per stage were evaluated. Also, n = 6 4T1 biopsy samples were assessed. All samples were evaluated in triplicate. MFIR: mean fluorescent intensity ratio. Ratio of membranous staining calculated from data reported in [15].

### Identification of individual cell populations

**Figure 3:**
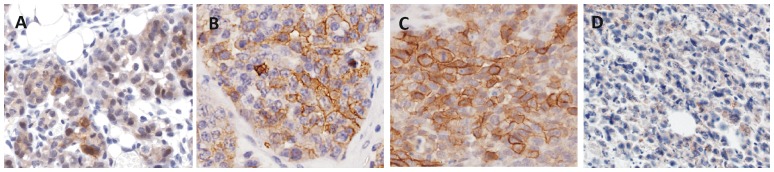
CXCR4 staining using immunohistochemistry. Membranous staining of fixed tumor tissue slices after incubation with the anti-CXCR4 antibody 2B11 in A) early, B) intermediate, C) late stage MIN-O tumor lesions and D) 4T1 control tumors (40× magnification).

**Figure 4:**
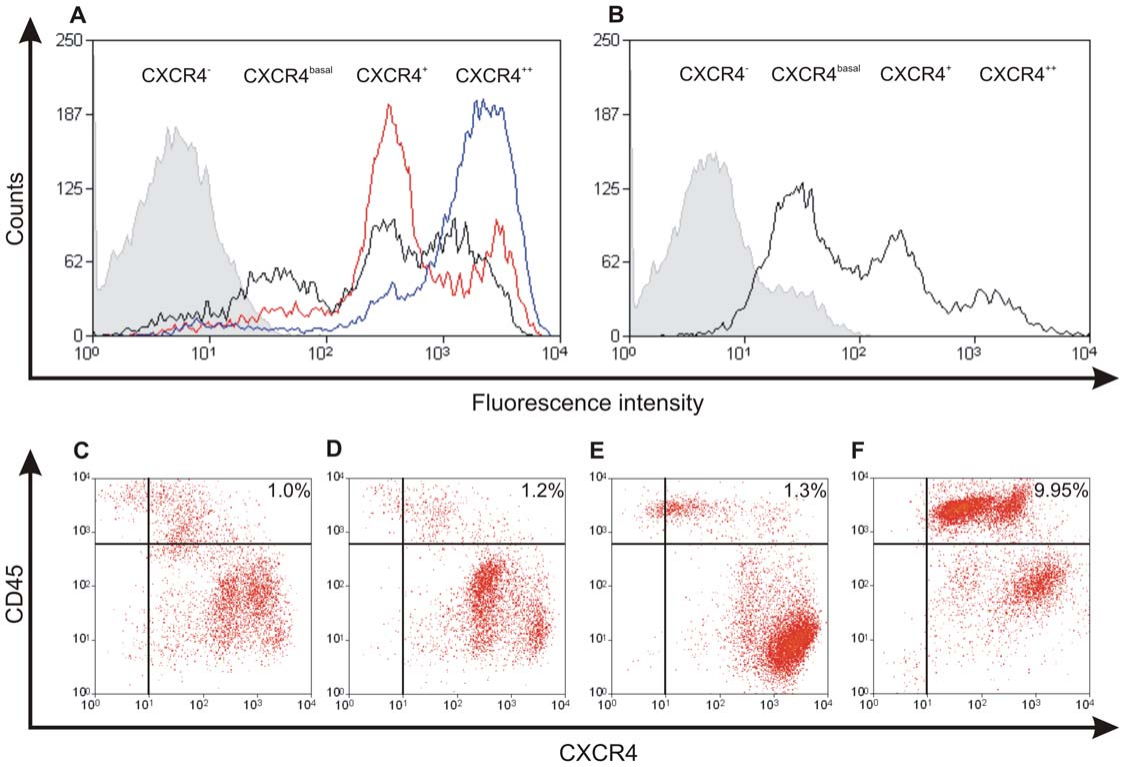
Fluorescence based fresh tumor biopsy analysis after incubation with MSAP-Ac-TZ14011. A) CXCR4 staining pattern in early (black), intermediate (red) and late stage (blue) MIN-O biopsy samples. B) CXCR4 staining pattern in 4T1 biopsy samples. Non-incubated control samples are depicted in grey. C–F) Analysis of CD45 expression in CXCR4 positive cells in early, intermediate and late stage MIN-O tumor lesions and 4T1 tumor lesions. For percentages of populations with different CXCR4 expression, see Table 2.

IHC revealed increasing but heterogeneous membranous staining for CXCR4 in the MIN-O lesions (Table 1, Fig. 3). Flow cytometric analysis enabled identification of different CXCR4 expressing cell populations after incubation with MSAP-AcTZ14011^.^(Fig. 4). As incubation occurred at 4°C, these populations were differentiated based on the cell membrane associated binding of the imaging agent. Fig. 4A shows that besides a low percentage of CXCR4 negative cells (CXCR4^−^ mean 13.2±2.6), three distinct populations were evident; CXCR4^basal^ (mean 58.9±3.7), CXCR4^+^ (mean 367.3±47.0) and CXCR4^++^ (mean 2197.4±413.3).

The different cell populations were found in the MIN-O lesions as well as in the 4T1 tumor specimens. The increase in fluorescence intensity between the different populations was determined by calculating the MFIR between the CXCR4^basal^ and the CXCR4^+^ or CXCR4^++^ population. This resulted in a 7.8±1.2-fold higher ratio in the CXCR4^+^ and a 47.0±10.4-fold higher ratio in the CXCR4^++^ population compared to the CXCR4^basal^ population. Carlisle *et al.* [26] reported comparable differences in fluorescent intensities when comparing several cell lines with different levels of CXCR4 expression. As such it appears that during tumor progression different CXCR4 positive cell populations exist within the tumor.

Further analysis of the results obtained with flow cytometry revealed that the percentage of CXCR4^basal^ cells was highest in the 4T1 tumor samples and that in the MIN-O lesions this percentage of cells decreased during lesion progression (Table 2). The percentage of strongly CXCR4 positive (CXCR4^+^ and CXCR4^++^) cells increased from 68.6±1.5% in early stage MIN-O lesions to 86.6±0.9% and 93.0±0.7% in intermediate and late stage MIN-O lesions, respectively (Table 2). Furthermore, the percentage of strongly CXCR4 positive cells was significantly higher in all stages of MIN-O progression compared to the percentage found in the control 4T1 tumor lesions (44.6±6.0%; p<0.001).

**Table 2:** Staining percentages of populations with different CXCR4 expression.

	% CXCR4^−^	% CXCR4^basal^	% CXCR4^+^	% CXCR4^++^	% CXCR4^+/++^
MIN-O (early stage)	6.9±0.8	25.9±0.8	38.4±2.3	30.3±2.0	68.6±1.5
MIN-O (intermediate stage)	2.9±0.3	11.4±0.7	53.5±2.8	33.1±3.2	86.6±0.9
MIN-O (late stage)	3.0±0.5	4.7±0.5	12.9±1.0	80.0±1.4	93.0±0.7
4T1	1.2±0.4	56.1±5.3	27.0±2.3	18.3±4.3	44.6±6.0

In the MIN-O lesions the percentage CXCR4^+^ and CXCR4^++^ cells varied during tumor progression (Fig. 4A). In the early stage MIN-O lesions 38.4±2.3% of the cells was CXCR4^+^ and 30.3±2.0% of the cells was CXCR4^++^. Intermediate stage lesions showed a similar expression pattern, however, the percentage of CXCR4^+^ cells increased to 53.5±2.8%, whereas in late stage lesions the percentage of CXCR4^+^ cells had decreased to 12.9±1.0%. A 2.5-fold (range 2.4–2.6) increase in CXCR4^++^ cells could be seen in the late stage lesions when compared to the intermediate and early stage lesions (80.0±1.4% vs. 33.1±3.2% and 30.3±2.0%, respectively) (Table 2).

### Identification of the amount of CXCR4 expressing lymphocytes

CXCR4 is not only expressed by tumor cells, but can also be expressed by native immune cells such as lymphocytes [27]. As the latter can also be present in tumor lesions [19], a control staining for lymphocytes to exclude over- or underestimation of the amount of CXCR4 positive tumor cells is required. Co-incubation with both MSAP-Ac-TZ14011 and an anti-CD45 antibody were used to determine the amount of lymphocytes (CD45^+^) that were CXCR4 positive. Flow cytometric analysis revealed that the percentage of CD45^+^ lymphocytes was highest in the 4T1 tumor cell suspensions (9.95%). On the contrary, in the MIN-O lesions, the amount of CD45^+^ lymphocytes was very low, and only increased slightly from 1.0% in early stage to 1.2% in intermediate and 1.3% in late stage lesions.

Co-staining could be used to specify which CXCR4 positive populations contained the CD45^+^ cells (Fig. 4C–F) by differentiating between the different CXCR4 expressing cell populations (x-axis) and the CD45 expression of the cells (y-axis). In the MIN-O lesions concomitant staining between CXCR4 and CD45 was mainly seen in the CXCR4^basal^ population (Fig. 4C–E) whereas in the 4T1 controls CD45^+^ cells were predominantly present in the CXCR4^basal^ and CXCR4^+^ populations (Fig. 4F). Although clearly detectable, the percentages of CD45^+^ cells found in the different MIN-O and 4T1 tumor tissue samples are not likely to influence the CXCR4 based discrimination between the MIN-O and the 4T1 tumor lesions.

### Fluorescence IHC of CXCR4 expression

**Figure 5:**
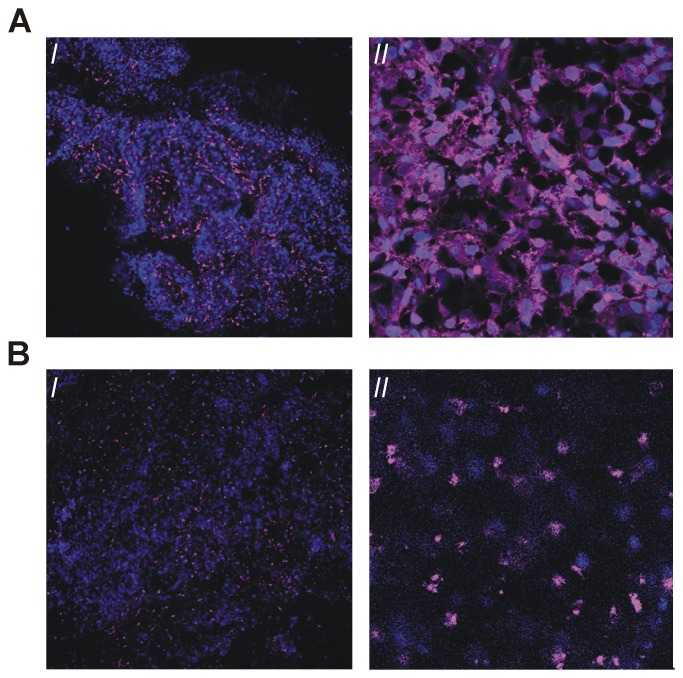
Evaluation of freshly isolated MIN-O tumor slices. A) *ex vivo* incubation with MSAP-Ac-TZ14011 and B) after intravenous injection of MSAP-Ac-TZ14011 twenty-four hours prior to evaluation of the tissue. I: 10× magnification. II) 63× magnification. Signal emitted by MSAP-Ac-TZ14011 is depicted in magenta and DAPI (blue) was used to visualize the cell nucleus.

**Figure 6:**
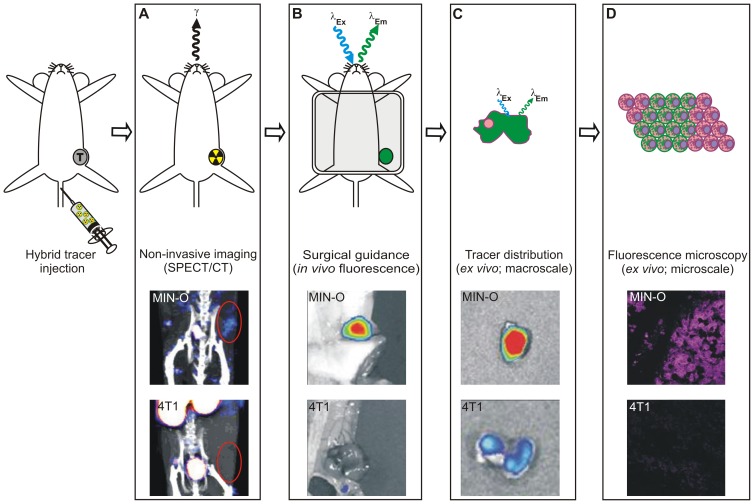
Non-invasive imaging. A) SPECT/CT imaging after intravenous injection of ^111^In-MSAP-Ac-TZ14011. B) *in vivo* and C) *ex vivo* fluorescence imaging. D) fluorescence microscopy (10× magnification). Intermediate stage MIN-O: top images. Late stage 4T1: bottom images.

Similar to Ac-TZ14011-FITC [17], MSAP-Ac-TZ14011 could also be used during fluorescence IHC applications. Confocal microscopy at 37°C after *ex vivo* incubation (at 4°C) of fresh tumor slices revealed both membranous and cytoplasmic staining throughout the late stage MIN-O tumor lesion (Fig. 5A I and 5A II). In the 4T1 control lesions hardly any fluorescence staining could be observed under the same conditions. Accumulation in 4T1 tumor lesions was comparable to the image in Figure 6D.

### *In vivo* SPECT/CT and fluorescence imaging

Flow cytometric and fluorescence IHC data combined allowed an accurate differentiation between predominantly CXCR4-basal 4T1 tumors and CXCR4-positive MIN-O lesions. *In vivo*, MIN-O lesions characterized as mainly CXCR4^+^/CXCR4^++^ at initial screening (see above) could be accurately identified with SPECT/CT imaging after intravenous injection of ^111^In labeled MSAP-Ac-TZ14011 (^111^In-MSAP-Ac-TZ14011). The 4T1 tumor lesions showed no tracer accumulation at the same imaging settings (Fig. 6A).

Evaluation of the ^111^In-MSAP-Ac-TZ14011 uptake levels in the tumor lesions (%ID/g) resulted in a tumor-to-muscle ratio that was 3.8 times higher in the late stage MIN-O lesions (4.55±0.67) as compared to the 4T1 lesions (1.20±0.12; Table 3). Biodistribution for both models was conform previously reported results [15]. Quantification of the fluorescence signal intensities in these tumor lesions revealed results comparable to the radioactivity measurements (Table 3). The signal intensity was 4.2 times higher in the late stage MIN-O lesions compared to the 4T1 tumor lesions (1.09×10^9^±1.7×10^8^ vs. 2.5×10^8^±3.8×10^7^ photons/sec/cm^2^ respectively) (Fig. 6B). This 4-fold difference seen with both SPECT/CT and fluorescence imaging was in accordance with the differences found in membranous CXCR4 expression (Table 1).

**Table 3:** In vivo imaging: Ratio between signal in late stage MIN-O and 4T1 tumor lesions.

	Ratio signal in MIN-O/ 4T1
Radioactivity (%ID/g)	3.8
Fluorescence (photons/sec/cm^2^)	4.2

### *Ex vivo* assessment of tracer distribution

Although conventional IHC still acts as “golden standard” for the ex vivo evaluation (see Table 1), the fluorescent labels can also be detected ex vivo using a fluorescence microscope. *Ex vivo* fluorescence assessment of the tracer distribution in fresh tumor tissue segments following the systemic injection of MSAP-Ac-TZ14011 (24 hours prior to tumor excision) predominantly revealed accumulation of the imaging agent in the cytoplasm of the MIN-O lesions (Fig. 5A I and II and Fig. 6D), something that was not seen in the 4T1 control samples (Fig. 6D). Cytoplasmic staining found is in line with the internalization of CXCR4 receptors over time at 37°C [17].

Compared to systemic tracer administration, differences in staining patterns were observed after direct (*ex vivo*) incubation of tumor tissue samples with MSAP-Ac-TZ14011 (Fig. 5A) (Fig. 4B). Direct, *ex vivo*, incubation of the tumor tissue will probably enable visualization of “all” CXCR4 positive cells present, systemic administration will most certainly only stain cells that could be reached by the tracer via the vascular network.

Evaluation of the α_ν_β_3_-integrin expression in late stage MIN-O lesions and 4T1 tumor lesions previously revealed that the degree of angiogenesis in both MIN-O and 4T1 tumor lesions is similar [19]. The lesions are overall well perfused, but some regions contained more and larger blood vessels than others. This heterogeneity in the vascular physiology will likely be of influence on the distribution of the tracer throughout the tumor.

## Discussion

Imaging applications using hybrid tracers are rapidly emerging [28]–[31] and have already been successfully applied in clinical studies facilitating integrated pre- and intraoperative imaging of sentinel nodes [10]–[15]. By adding a receptor targeting moiety, the utility of hybrid imaging agents can be expanded to pre-imaging screening of biomarker expression levels and subsequent (imaging) target selection.

In a previous comparison of currently available imaging agents for CXCR4, Ac-TZ14011 showed great potential in fluorescence imaging and hybrid imaging applications [17]. Ac-TZ14011 was shown to bind selectively to CXCR4 and could be used to visualize CXCR4 positive tumor lesions *in vivo* [18], [19], [21], [22], [32]. It must be noted that MSAP-Ac-TZ14011 derivatives are currently the only hybrid imaging agents available for CXCR4 targeting [17]. For *in vivo* imaging experiments with this tracer the use of a relatively low specific activity was shown to be beneficial for tumor visualization [17]. We have previously demonstrated that tumor models, which more accurately represent the modest five-fold CXCR4 over-expression found in the clinical situation, such as the MIN-O model used in this study, better represent the clinically found CXCR4 expression levels in tumors [17]. As a result the *in vivo* SPECT/CT images obtained in this study (Figure 6) provide less of a black and white discrimination between CXCR4 positive tumors and their background, than can be obtained using transfected tumor cells with extremely high levels of CXCR4 expression [17].

The hybrid nature of the CXCR4 targeting imaging agent MSAP-Ac-TZ14011 has allowed us to successfully demonstrate the concept of integrating target selection in fresh (biopsy) tumor tissue with *in vivo* imaging and *ex vivo* microscopic validation (Fig. 1), all using a single imaging agent.

Similar to the *in vitro* evaluation of fluorescently labeled imaging agents [21], [22], flow cytometriy could be used to analyze the level of CXCR4 expression in the tumor cell suspensions. In this application incubation with MSAP-Ac-TZ14011 allowed effective discrimination between MIN-O (stages) and 4T1 tumor lesions using the fluorescent label of the targeting hybrid imaging agent (Fig. 4 and Table 2). These findings were further confirmed with IHC (Table 2), *in vivo* SPECT/CT (using the radiolabel) and fluorescence imaging (Fig. 6); the ratio between the late stage MIN-O and similarly sized 4T1 control lesions was comparable with all visualization methods.

Flow cytometry is already being used in a clinical setting for applications such as diagnosis of leukemia and lymphoma [33]–[36] and dependent on the analyzer used, flow cytometry enables assessment of up to 15 cell surface parameters in one sample [35]. Even the small early stage tumors (<100 mm^3^), which are comparable in size with human biopsy samples, contain sufficient cells for characterization of multiple samples. In this study co-incubation of MSAP-Ac-TZ14011 and an anti-CD45 antibody showed that at least two markers could be simultaneously evaluated in fresh tumor specimens and that the presence of native immune cells in the tumor tissue could be assessed. Addition of such a control staining can be used to exclude over- or underestimation of tumor-cell related biomarker expression levels. For future applications in cancer management it will be possible to set up such screens using a number of biomarker targeting imaging agents simultaneously. By labeling each agent with a different fluorescent dye a tailored selection of the most prominently available receptor proteins suitable for imaging can then be made. The method of staining and the fact that perfusion is essential for good visualization *in vivo* should, however, be taken into account when comparing flow cytometry/IHC to *in vivo* imaging results.

In agreement with the results obtained with flow cytometric analysis after incubation with MSAP-Ac-TZ14011, we have previously demonstrated that CXCR4 expression in MIN-O lesions is heterogeneous and that the degree of membranous staining of CXCR4 at IHC increases with lesion progression [19]. This increase in membranous staining was in concordance with the increase in uptake of ^111^In-Ac-TZ14011 during MIN-O lesion progression [19]. With IHC only total staining percentages can be obtained, whereas flow cytometry can also be used for the accurate evaluation of cell populations with different expression levels within one tumor sample (Table 1; [26]). As fluorescence intensities vary according to receptor expression on the cells, the (semi-quantitative) signal intensity levels can be directly, linked to receptor CXCR4 expression levels [37]. Flow cytometric analysis after incubation of the tumor cell suspensions with MSAP-Ac-TZ14011 underlined that CXCR4 positivity increased during the progression of MIN-O lesions. An increase in the percentage of CXCR4^+^ cells seemed to mark the transition into intermediate stage lesions. Concurrently, invasive late stage lesions mainly contained CXCR4^++^ cells, which is in line with the clinically reported higher expression of CXCR4 in more invasive types of breast cancer [6].

One can envision that besides the ability to select the most appropriate targeting imaging procedure, the level of over-expression of a biomarker that is associated with e.g. malignancy of a tumor [8], [38], [39], may also influence clinical decision-making. For example, CXCR4 expression is linked to a higher tendency to metastasize and higher levels of CXCR4 expression have been found in (distant) metastasis compared to the primary tumor [38]. It has also been proposed that CXCR4 expression levels can be used to select subsets of tumor lesions that show a more aggressive biological behavior [40]. Furthermore, Chu *et al.* [39] previously proposed that CXCR4 expression levels allow for the identification of subsets of patients who are at risk of developing recurrent disease, even within patient groups with an initial good prognosis.

It will be interesting to investigate what the influence of the presence of CXCR4^++^ cells will be on metastatic ability of metastatic MIN-O tumor models [23]. Herein the orthotopic MIN-O transplantation model used in this study [24], [41] serves as an ideal model; besides the evaluation of tumor progression into an invasive phenotype, MIN-O strains that metastasize have also been described [23].

The main goal of this study was to evaluate the concept of using biopsy tissue specimens for a personalized selection of the most optimal targeted imaging approach. Obviously, the concept described above is not limited to the targeting of CXCR4. A hybrid version of an imaging agent targeting a biomarker of choice can be used. Possibly, also a cocktail of imaging agents can be used for the simultaneous assessment of several markers at once. Use of different fluorescent dyes and/or radioactive isotopes will then enable discrimination between biomarkers. In this way, the screening and *in vivo* imaging data can still be used for accurate staging of the tumor lesions. The latter will not be possible when identically labeled imaging agents are used.

## Conclusions

Hybrid imaging agents can be used during the different steps encountered in the clinical management of cancer. Comparable quantitative results have been obtained during target selection in biopsy tissue (flow cytometry), *in vivo* imaging (SPECT/CT and fluorescence imaging) and during pathological validation (*ex vivo* microscopy) of the surgically excised tissue (*ex vivo* microscopic analysis). Incubation with MSAP-Ac-TZ14011 enabled accurate staging of MIN-O lesion progression via the CXCR4 expression pattern of the lesions. Although only CXCR4 was used in this proof of concept study, this approach can readily be expanded to other targeting hybrid imaging agents and will help increase the clinical applicability of tumor specific imaging approaches.
